# Using Pharmacokinetic Modeling and Electronic Health Record Data to Predict Clinical and Safety Outcomes after Methylprednisolone Exposure during Cardiopulmonary Bypass in Neonates

**DOI:** 10.32604/chd.2023.026262

**Published:** 2023-06-09

**Authors:** Henry P. Foote, Huali Wu, Stephen J. Balevic, Elizabeth J. Thompson, Kevin D. Hill, Eric M. Graham, Christoph P. Hornik, Karan R. Kumar

**Affiliations:** 1 Department of Pediatrics, Duke University, Durham, USA; 2 Duke Clinical Research Institute, Durham, USA; 3 Department of Pediatrics, Medical University of South Carolina, Charleston, USA

**Keywords:** Neonates, cardiopulmonary bypass, methylprednisolone exposure

## Abstract

**Background:**

Infants undergoing cardiac surgery with cardiopulmonary bypass (CPB) frequently receive intraoperative methylprednisolone (MP) to suppress CPB-related inflammation; however, the optimal dosing strategy and efficacy of MP remain unclear.

**Methods:**

We retrospectively analyzed all infants under 90 days-old who received intra-operative MP for cardiac surgery with CPB from 2014–2017 at our institution. We combined real-world dosing data from the electronic health record (EHR) and two previously developed population pharmacokinetic/pharmacodynamic models to simulate peak concentration (Cmax) and area under the concentration-time curve for 24 h (AUC24) for MP and the inflammatory cytokines interleukin-6 (IL-6) and interleukin-10 (IL-10). We evaluated the relationships between post-operative, safety, and other clinical outcomes obtained from the EHR with each predicted exposure using non-parametric tests.

**Results:**

A total of 142 infants with median post-natal age 8 (interquartile range [IQR]: 5, 37) days received a total dose of 30 (19, 49) mg/kg of MP. Twelve (8%) died, 37 (26%) met the composite post-operative outcome, 114 (80%) met the composite safety outcome, and 23 (16%) had a major complication. Predicted median Cmax and AUC24 IL-6 exposure was significantly higher for infants meeting the composite post-operative outcome and those with major complications. Predicted median Cmax and AUC24 MP exposure was significantly higher for infants requiring insulin. No exposure was associated with death or other safety outcomes.

**Conclusions:**

Pro-inflammatory IL-6, but not MP exposure, was associated with post-operative organ dysfunction, suggesting current MP dosing may not adequately suppress IL-6 or increase IL-10 to impact clinical outcomes. Prospective study will be required to define the optimal exposure-efficacy and exposure-safety profiles in these infants.

## Graphical Abstract

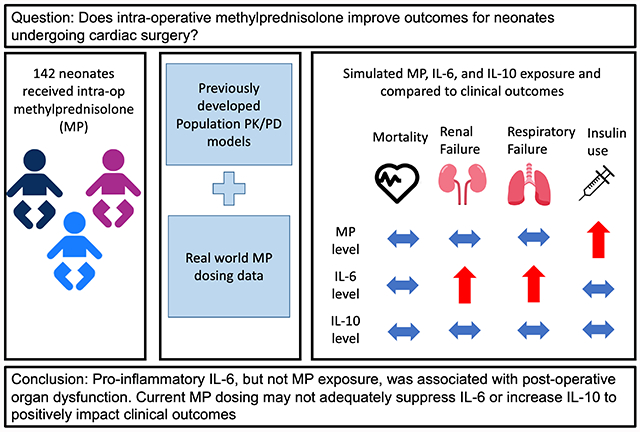

## 1 Introduction

Infants with congenital heart disease may undergo surgical palliation or correction in the neonatal period, which often requires the use of cardiopulmonary bypass (CPB). However, CPB can result in significant untoward physiologic effects and poor outcomes in this vulnerable population [1]. CPB can induce substantial systemic inflammation through the action of several cytokines including proinflammatory interleukin-6 (IL-6) and anti-inflammatory interleukin-10 (IL-10), and higher levels of inflammation correlate with worse post-operative outcomes [1–3]. More than 60% of neonates undergoing CPB are treated with perioperative methylprednisolone (MP) to suppress this inflammation, but the optimal dosing strategy and efficacy of MP remain unclear [4–9]. CPB can significantly alter drug disposition in infants, and limited data exist on the exposure-efficacy and exposure-safety profile of infants undergoing CPB exposed to MP, potentially leading to inadequate dosing and resultant therapeutic failures or toxicities [10–12].

Population pharmacokinetic/pharmacodynamic (PK/PD) models allow for studying drug disposition from sparse sampling methods. PK/PD studies typically have too few patients to precisely evaluate rare events, but these models can be combined with real-world dosing data to predict drug exposure in larger samples sizes, as well as assess the relationship between simulated exposure and efficacy and safety outcomes [13–15]. We previously developed two population PK/PD models for MP in infants undergoing CPB (12). These models simulate both MP drug exposure and biomarker surrogates for MP efficacy, IL-6, and IL-10. In this study, we combine the existing PK/PD models of MP in infants on CPB with real-world dosing and clinical data derived from the electronic health record (EHR) to evaluate the relationship between predicted exposures and both post-operative outcomes and adverse safety events for infants after MP exposure following CPB.

## 2 Patients and Methods

### 2.1 Study Design and Data Source

We performed a retrospective observational cohort study of all infants who underwent cardiac surgery at Duke University Medical Center between January 01, 2014 and December 31, 2017. We used the Duke Congenital Cardiothoracic Surgery Database, the Duke Enterprise Data Unified Content Explorer (DEDUCE), and Epic Clarity to identify the cohort and obtain data. The Duke congenital cardiothoracic surgery database includes information on all cardiac surgeries performed on children in accordance with the data collection process for the Society of Thoracic Surgeons (STS) Congenital Heart Surgery Database [16]. DEDUCE is a self-service web-based EHR query system [17]. Epic Clarity is a Microsoft SQL Server database that contains a large subset of EHR data extracted from the Duke Maestro Care (Epic) application. We obtained institutional review board approval from the Duke University Health System IRB with waiver of informed consent for our study, protocol Pro00104737.

### 2.2 Study Population

We included all infants who underwent cardiac surgery with CPB between postnatal age (PNA) 0 to 90 days and received intraoperative MP during CPB. For infants with multiple cardiac surgeries during the study period, we only included the first surgery with CPB. We excluded infants with missing Risk-Adjusted Classification for Congenital Heart Surgery (RACHS-1) scores.

### 2.3 Data Collection and Study Outcomes

From the above data sources, we extracted demographics (gestational age, postnatal age at time of surgery, birth weight, sex, race, ethnicity), congenital conditions (noncardiac congenital syndrome, chromosomal abnormalities, and cyanotic congenital heart disease as coded in the STS database), preoperative risk factors as coded in the STS database (receipt of mechanical circulatory support and/or cardiogenic shock; respiratory disease as defined by bronchopulmonary dysplasia, single lung physiology, or need for mechanical ventilation; renal disease as defined by kidney dysfunction and renal failure requiring dialysis, and systemic infection as defined by sepsis or RSV infection), operative variables (MP dosing, age at surgery, RACHS-1 score, The Society of Thoracic Surgeons-European Association for Cardio-Thoracic Surgery (STAT) mortality category, CPB time, cross-clamp time, and circulatory arrest time), and post-operative variables (receipt of post-operative steroids, post-operative length of stay, and hospital length of stay) [18–20].

Post-operative outcomes included death, defined as death before discharge, but not within 36 h post-operatively. We excluded death prior to 36 h post-operatively since these were considered less likely to be affected by methylprednisolone administration. Additional post-operative outcomes were as defined by the STS database: the need for post-operative mechanical circulatory support, defined as intra-aortic balloon pump, ventricular assistant device, extracorporeal membrane oxygenation, or cardiopulmonary support; peri-operative cardiac arrest, defined as unexpected perioperative (intraoperative and post-operative) cardiac arrest; cardiac dysfunction or failure defined as severe cardiac dysfunction or low cardiac output syndrome; respiratory failure, defined as mechanical ventilator support >7 days, reintubation, or need for tracheostomy; renal failure, defined as need for dialysis or hemofiltration post-operatively or at the time of discharge; or the composite outcome of any of death, mechanical circulatory support, cardiac arrest, cardiac dysfunction/failure, respiratory failure, or renal failure [16].

Safety outcomes included the need for any insulin within 24-h post-operatively; maximum serum glucose within 24-h post-operatively; hyperglycemia, defined as serum glucose ≥200 mg/dL, within 24-h post-operatively; poor wound healing defined as sterile wound dehiscence or sternal instability; infection defined as pneumonia, sepsis, endocarditis, or wound infection (superficial, deep, or mediastinitis); or composite safety outcome of any of insulin requirement, hyperglycemia, poor wound healing, or infection.

Major complications were based off the definition by the STS database as renal failure requiring dialysis, neurologic deficit persisting at discharge, arrhythmia requiring permanent pacemaker, paralyzed diaphragm/phrenic nerve injury, mechanical circulatory support, and unplanned surgical or catheter-based cardiac and non-cardiac re-interventions [21]. Operative mortality was defined as death during hospitalization even after 30 days or before the end of the 30^th^ post-operative day if discharged from hospital.

### 2.4 Exposure Simulations

The published population PK/PD models that were used to simulate MP, IL-6, and IL-10 exposure were based on a two-compartment model with first-order formation rate to MP and estimation of the allometric coefficient on CL [12]. The models incorporated both a single intraoperative MP dose at the time of CPB, as well as a split dosing regimen including intraoperative and pre-operative MP prescribed for CPB-related inflammation. None of the models included post-operative steroid administration. The previously developed PK/PD models for MP, IL-6, and IL-10 were based on infants who received either a single intraoperative MP dose or a two-dose regimen with an additional MP dose ten hours preoperatively. The model parameters allowed for simulated MP, IL-6, and IL-10 exposure for the varying real-world dosing regimens of the infants included in the current study. The final model for MP clearance and volume of distribution included CPB time (CPBtime) and weight (WT) as significant covariates for CL according to Eq. (1):

(1) $$CL=\left[3.88*\left(1-POSTCPB\right)+3.88*POSTCPB*{\left(\frac{CPB_{time}}{156.6}\right)}^{-0.47}\right]*{\left(\frac{WT}{3.2}\right)}^{1.24}$$

where POSTCPB is time after CPB in hours. The apparent volume of distribution for central compartment (Vc) was estimated as 8.92 L and apparent volume of distribution for peripheral compartment (Vp) was estimated as 16.81 L, both centered to a 3.2 kg median weight for the original model development cohort.

As described previously, change in IL-6 over time was best described by an indirect response model with partial interaction between CPB effect and drug effect, with RACHS-1 score as a significant covariate for the CBP effect. For change in IL-10 over time, an indirect response model with complete interaction between CPB effect and drug effect provided the best fit to the data, and post-menstrual age was a significant covariate for the CPB effect (see Appendix A for complete description of equations). We used the nonlinear mixed-effect modeling software NONMEM and simulated concentrations of MP, IL-6, and IL-10 at both maximum (Cmax) and integrated concentration over 24 h post-operatively (AUC24).

### 2.5 Statistical Analysis

We used standard summary statistics to describe infant characteristics including counts (with percentages) for categorical variables and medians (with interquartile values) for continuous variables. We visually compared the logarithmic distribution of MP, IL-6, and IL-10 Cmax and AUC24 simulated exposure predictors by each categorical outcome using standard box-and-whisker plots and by each continuous outcome using scatter plots. We non-parametrically compared MP, IL-6, and IL-10 Cmax and AUC24 simulated exposure predictors by each categorical post-operative and adverse safety outcome using the Wilcoxon rank-sum test. We defined statistical significance as a *p*-value < 0.05. We made no adjustment for multiple comparisons because a large number of the study outcomes are not independent and have a high degree of correlation. We performed all statistical analyses using Stata 17.0 (College Station, Texas).

## 3\ Results

### 3.1 Patient Characteristics

We identified 142 infants who met study inclusion and exclusion criteria (Table 1).

We excluded five infants with missing RACHS-1 scores from analysis. Overall, the infants had a median (IQR) gestational age of 39 weeks (38, 39) and median birth weight of 3170 g (2720, 3600). Seventy-nine (56%) infants had cyanotic congenital heart disease and the median RACHS-1 score was 4 (3, 4). The median age at surgery was 8 (5, 37) days. We found that prior to surgery, 15 (11%) infants had cardiogenic shock, 3 (2%) required mechanical circulatory support, 72 (51%) had underlying respiratory disease, 4 (3%) had renal dysfunction, and 4 (3%) had systemic infection. All infants received intraoperative MP and 134 (94%) received additional preoperative MP with a median time between MP doses of 6 h (5, 10). The median total MP dose received preoperatively and intraoperatively was 30 mg/kg (19, 49) across a median of 2 doses (2, 3). A total of 13 (9%) infants received additional steroids post-operatively on median postoperative day 7 (4, 29).

### 3.2 Simulated Exposures

Across all infants, the median predicted MP Cmax and AUC24 were 2 mcg/mL (2, 4) and 25 mcg • h/mL (16, 43) (Appendix B). The median predicted IL-6 exposure was Cmax 117 pg/mL (56, 145) and AUC24 2056 pg • h/mL (962, 2380). The median predicted IL-10 exposure was Cmax 180 pg/mL (111, 299) and AUC24 793 pg • h/mL (441, 1206).

### 3.3 Post-Operative Outcomes

Twelve (8%) infants died, 37 (26%) met the composite post-operative outcome, and 23 (16%) had a major STS complication (Table 2).

Eleven (8%) required mechanical circulatory support, 6 (4%) had cardiac arrest, 5 (4%) had cardiac dysfunction, 19 (13%) developed respiratory failure, and 3 (2%) developed renal failure.

**Figure 1:**
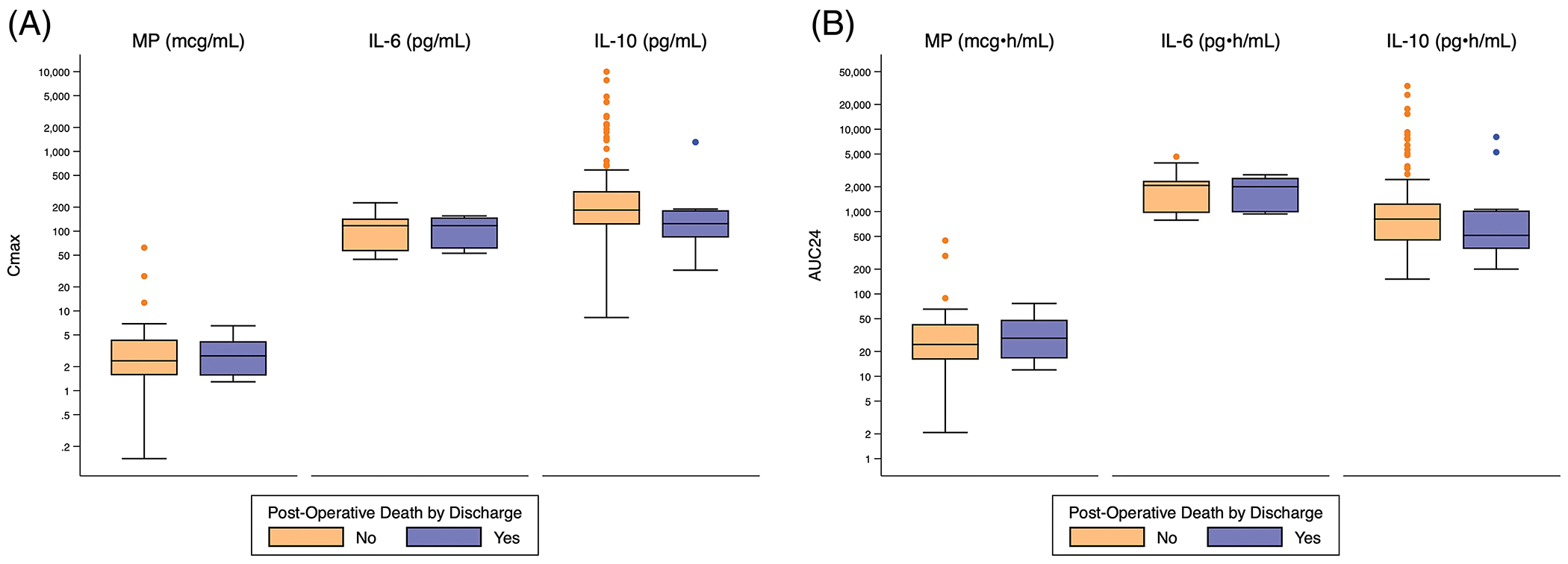
Distribution of Cmax and AUC24 exposure predictors based on post-operative death. Standard box-and-whisker plots demonstrating the logarithmic distribution of MP, IL-6, and IL-10 simulated exposure predictors by post-operative death for: (A) Cmax; and (B) AUC24. AUC24 = area under the concentration time curve for 24 h; Cmax = peak concentration; IL-6 = interleukin-6; IL-10 = interleukin-10

There was no significant difference in exposure predictors for death or operative mortality (Fig. 1, Appendix C, Appendix D).

**Figure 2:**
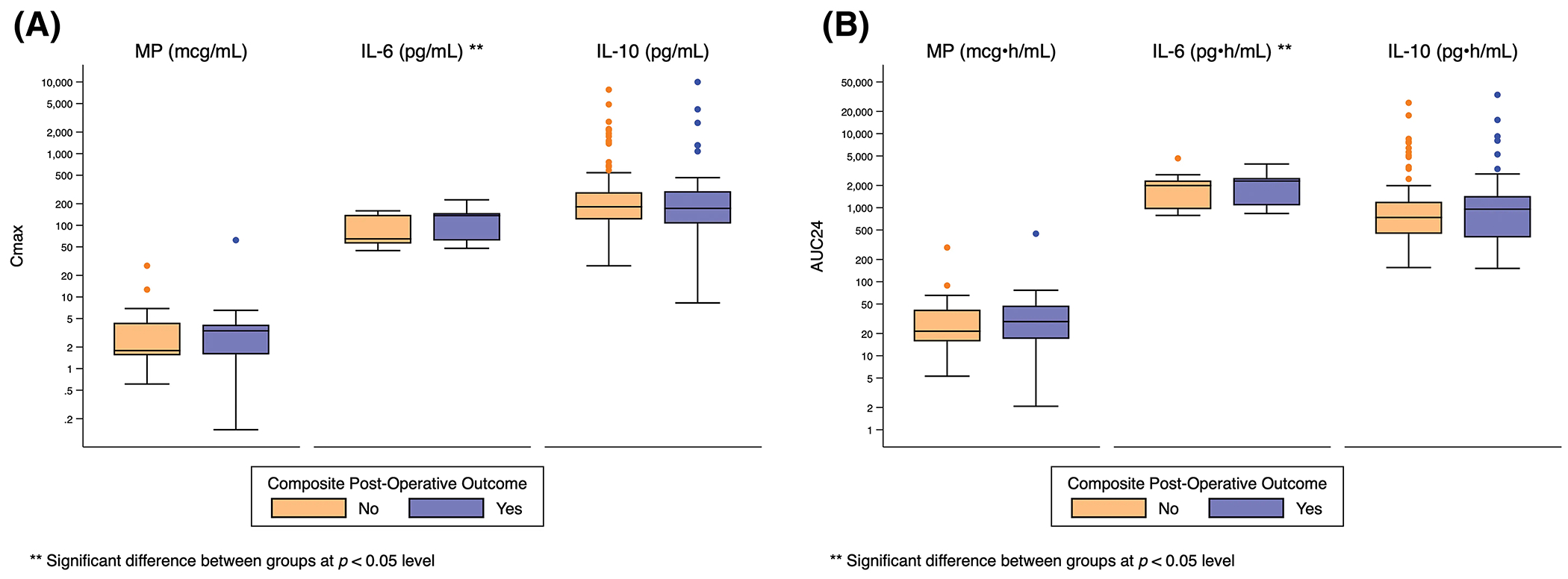
Distribution of Cmax and AUC24 exposure predictors based on composite post-operative outcome. Standard box-and-whisker plots demonstrating the logarithmic distribution of MP, IL-6, and IL-10 simulated exposure predictors by composite post-operative outcome for: (A) Cmax; and (B) AUC24. AUC24 = area under the concentration time curve for 24 h Cmax = peak concentration; IL-6 = interleukin-6; IL-10 = interleukin-10

Both peak and total IL-6 exposure were significantly higher for infants who required post-operative circulatory support (Cmax: 141 pg/mL [126, 151] *vs*. 114 pg/mL [56, 144], *p* = 0.04; AUC24: 2351 pg • h/mL [2189, 2569] *vs*. 2030 pg • h/mL [959, 2358], *p* = 0.03), developed respiratory failure (Cmax: 143 pg/mL [118, 151] *vs*. 111 pg/mL [56, 144], *p* = 0.02; AUC24: 2356 pg • h/mL [1993, 2520] *vs*. 2012 pg • h/mL [957, 2343], *p* = 0.009), developed renal failure (Cmax: 155 pg/mL [145, 184] *vs*. 116 pg/mL [56, 144], *p* = 0.02; AUC24: 2799 pg • h/mL [2520, 3327] *vs*. 2035 pg • h/mL [961, 2358], *p* = 0.007), or met the composite primary outcome (Cmax 137 pg/mL [61, 148] *vs*. 65 pg/mL [56, 140], *p* = 0.03; AUC24: 2294 pg • h/mL [1075, 2520] *vs*. 1993 pg • h/mL [56, 2335], *p* = 0.004) (Fig. 2, Appendices D–H).

There was no significant relationship between exposure predictor levels for MP or IL-10 and any clinical outcome (Fig. 2, Appendices D–H).

### 3.4 Safety Outcomes

**Figure 3:**
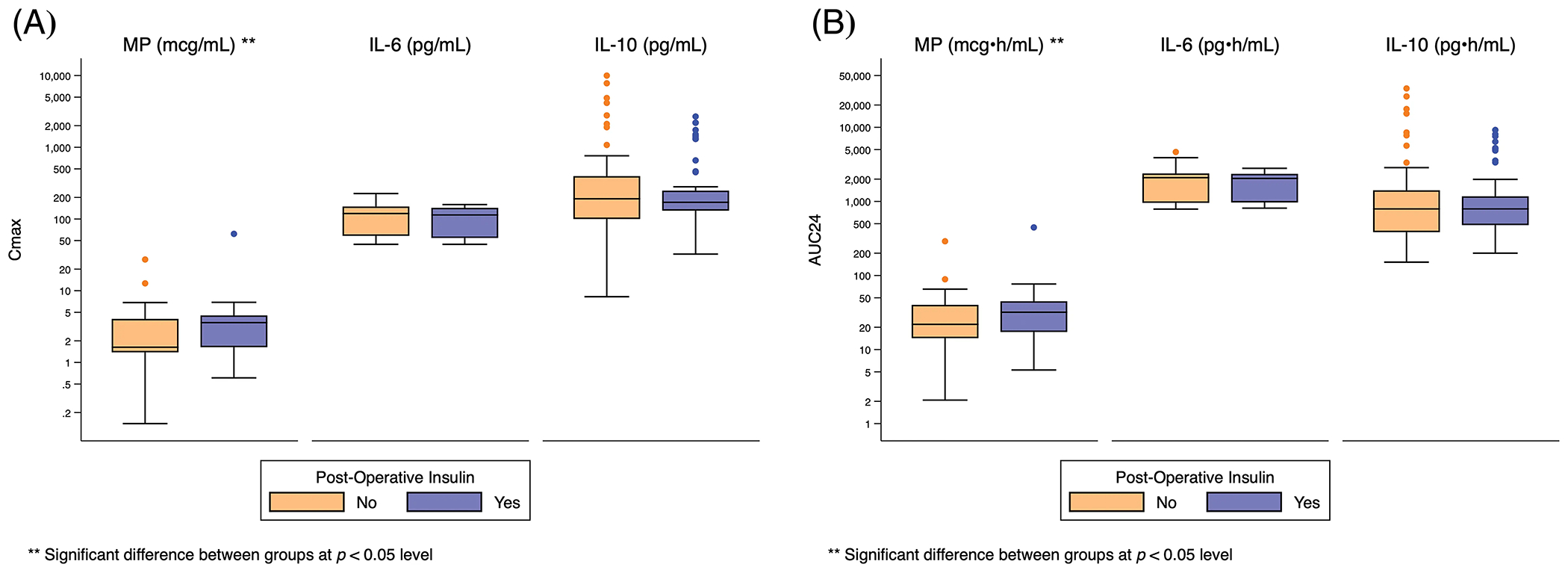
Distribution of Cmax and AUC24 exposure predictors based on post-operative insulin use. Standard box-and-whisker plots demonstrating the logarithmic distribution of MP, IL-6, and IL-10 simulated exposure predictors by post-operative insulin use for: (A) Cmax; and (B) AUC24. AUC24 = area under the concentration time curve for 24 h; Cmax = peak concentration; IL-6 = interleukin-6; IL-10 = interleukin-10

One hundred fourteen (80%) infants met the composite safety outcome (Table 2). Sixty-three (44%) required insulin therapy and 109 (77%) developed hyperglycemia. Five (4%) infants had poor wound healing and 4 (3%) developed infection. The need for insulin was significantly associated with higher peak and total MP exposure (Cmax: 4 mcg/mL [2, 5] *vs*. 2 mcg/mL [1, 4], *p* = 0.006; AUC24: 32 mcg • h/mL [17, 45] *vs*. 22 mcg • h/mL [14, 40], *p* = 0.02), but not IL-6 or IL-10 exposure (Fig. 3, Appendix I).

There was no significant relationship between exposure predictor levels for MP, IL-6, or IL-10 and hyperglycemia, poor wound healing, infection, or the composite safety outcome (Appendices I and J).

## 4 Discussion

Using real-world dosing and clinical information from the EHR combined with population PK/PD models in infants receiving MP during CPB for cardiac surgery, we found that predicted IL-6 exposure was associated with post-operative organ dysfunction and MP exposure was associated with higher insulin requirement but not improved clinical outcomes. These findings suggest that a therapeutic range for MP is not regularly obtained with current dosing strategies or that there may be no therapeutic benefit in this population.

CPB produces pro-inflammatory cytokines including IL-6 that are robustly linked to increased postoperative morbidity including cardiac dysfunction, renal injury, transfusion requirements, and prolonged intensive care unit stay [3,22–25]. Perioperative treatment with corticosteroid consistently improves inflammatory profiles, reducing IL-6 and other pro-inflammatory cytokine levels and increasing antiinflammatory IL-10 [6,9,26,27]. However, traditional clinical trials have not shown that corticosteroid therapy improves clinical outcomes in infants with congenital heart disease undergoing CPB, potentially due to suboptimal dosing [28–30]. It is challenging to ascertain the optimal exposure-response relationship of corticosteroids in this population, due in part to hesitancy to enroll this vulnerable population into randomized trials and barriers obtaining enough participants to adequately power doseranging studies [31,32]. Therefore, the appropriate therapeutic range in this population has not been well characterized. There is significant concern that current corticosteroid dosing may result in inadequate modulation of IL-6 and IL-10 levels and subsequent treatment failure or in toxicity from steroid-induced hyperglycemia or infection risk [7,33,34]. Our study design provides a combined evaluation of real-world drug exposure, biomarker response, and clinical endpoints that is challenging to achieve in a clinical trial and provides additional information of the exposure-efficacy and exposure-safety profiles for MP.

Our data do not demonstrate an association between predicted MP exposure and improved postoperative outcomes; however, we did find that infants that required post-operative mechanical support, developed renal or respiratory failure, and met the composite post-operative outcome had significantly higher predicted peak and total IL-6 exposure. This is consistent with previous reports that described an association between worse clinical outcomes and increased inflammatory markers, although no relationship with mortality was seen in our study [24,25,35]. Our data may indicate that current MP dosing strategies do not achieve adequate levels for effective modulation of IL-6. Alternatively, there may be a maximum effective exposure of MP that was achieved or exceeded by all infants in our cohort and provided a consistent baseline reduction in IL-6. Subsequently, the association of higher IL-6 exposure with worse clinical outcomes would suggest some infants had severe inflammation that exceeded the potential for amelioration by MP. A recent randomized controlled trial of neonates undergoing cardiac surgery found a significant site effect on the benefit of intra-operative hydrocortisone, supporting patient and peri-operative management factors play an important role in regulating inflammation and clinical outcomes [28]. Applying our model to cohorts treated with alternative MP dosing strategies that resulted in MP exposure in distinct ranges would provide further information on the dose-response curve.

Although our study demonstrated that peak and total MP exposure was associated with the need for postoperative insulin use in the first 24 h, there was no relationship between MP exposure and total rates of hyperglycemia. Overall, hyperglycemia was common in our cohort, occurring in 77% of infants. This suggests that current MP dosing practices may result in serum MP concentrations at the upper end of the exposure-response relationship with hyperglycemia. Alternatively, post-operative hyperglycemia may be predominantly driven by factors independent of exogenous corticosteroid, including the physiologic stress response to surgery, medications such as exogenous catecholamines, and relative insulin resistance in the neonatal population [36–38]. Significant adverse safety effects such as poor wound healing and infection were also not predicted by simulated exposure. The prevalence of these side effects was low in the overall cohort, occurring in only 4% and 3% of infants respectively, so it is possible that the analysis was underpowered to detect a relationship between exposure and the adverse events of poor wound healing or infection.

Our study has several strengths. Combining our previously developed population PK/PD models with clinical outcomes allows for correlation of simulated MP exposure and intermediary biomarker responses with clinical endpoints [12]. This analysis provides a more nuanced view of potential modifiers of clinical outcomes than is commonly ascertained with traditional trials focused on initial intervention and clinical effect. Within the observed range of IL-6 responses to current MP dosing, higher levels of IL-6 still correlate with worse clinical outcomes. This relationship suggests that efforts to further suppress IL-6 and the inflammatory response may allow for improved outcomes. Additionally, we used real-world dosing and clinical outcome data already collected in the EHR. This strategy allows for applying this analysis to a relatively large cohort of infants without the challenges of enrolling new subjects in a traditional trial [28,29]. Our approach can be applied to additional cohorts of infants, potentially as dosing practices change, to further assess the relationship between drug exposure, biomarker response, and clinical endpoints.

There are some limitations to our study. In our previously developed population PK/PD models, MP exposure is driven primarily by CPB time [12]. Patient weight with RACHS-1 score and post-menstrual age were included as significant covariates for the CPB effect on IL-6 and IL-10, respectively. The initial model relied on sparse sampling methods, especially during CPB, so obtained data points may not have fully described the time-concentration curves. Other covariates may be significant drivers of exposure but were not included in the model precluding full capture of dosing-exposure relationship in this population. Furthermore, since this was a single-center retrospective study, site-specific practices may be primary drivers in the assessed outcomes, which may constrain the generalizability of the findings. Even with these limitations, the distribution of predicted IL-6 exposure in this current study is similar to those measured directly during previous studies in post-CPB neonates treated with MP and lower than those seen for untreated infants, suggesting our simulations generated a plausible overall predicted exposure profile [6,34,39].

The optimal therapeutic concentration for MP, as well as therapeutic targets for IL-6 and IL-10 concentrations remain unknown, and treatment benefit from intra-operative MP remains unclear. This makes it challenging to define an ideal dose-exposure relationship. Our simulated data based on real-world dosing information demonstrates no association between MP exposure and surgical outcomes. An ongoing randomized, placebo-controlled, double-blind, multicenter trial seeks to assess the clinical benefit and safety of intraoperative MP [40]. This study may further clarify the utility of MP in improving care for these infants.

In this study, we combine developed population PK/PD models with real-world data from the electronic health record to predict exposures and assess outcome relationships. MP exposure predicted need for insulin, but not other adverse safety events. IL-6, but not MP exposure, predicted post-operative organ dysfunction. Current MP exposure may be inadequate for effective moderation of IL-6, IL-10, and the CPB-induced inflammatory response. Further study is needed to define optimal MP dosing to decrease IL-6, increase IL-10, and improved clinical outcomes while minimizing steroid-related toxicities.

## Supplementary Material

Appendix J

Appendix I

Appendix H

Appendix F

Appendix G

Appendix E

Appendix C

Appendix B

Appendix D

Appendix A

## Figures and Tables

**Table 1:** Infant and operative characteristics

Characteristic	N = 142
**Demographics**	
Gestational age (weeks)	39 (38, 39)
Birth weight (g)	3170 (2720, 3600)
Male	84 (59)
Race	
White	78 (55)
Black	39 (27)
Other	25 (18)
Hispanic ethnicity	13 (9)
Noncardiac congenital syndrome or chromosomal	37 (26)
abnormality	
Cyanotic congenital heart disease	79 (56)

**Pre-operative risk factors**	
Pre-operative length of stay (days)	7 (4, 10)
Mechanical circulatory support	3 (2)
Cardiogenic shock	15 (11)
Respiratory^a^	72 (51)
Renal^a^	4 (3)
Infectious^a^	4 (3)

**Operative variables**	
Total methylprednisolone dose (mg/kg)	30 (19, 49)
Total methylprednisolone dose (mg/kg/dose)	12 (10, 17)
Number of methylprednisolone doses	2 (2, 3)
Methylprednisolone exposure duration (hours)	6 (5, 10)
Age at surgery (days)	8 (5, 37)
Postmenstrual age at surgery (weeks)	40 (40, 42)
RACHS-1 score	4 (3, 4)
STAT mortality category	
1	10 (7)
2	24 (17)
3	23 (16)
4	62 (44)
5	23 (16)
Cardiopulmonary bypass time (minutes)	181 (126, 253)

**Post-operative variables**	
Post-operative steroids	13 (9)
Operative mortality^b^	12 (8)
Post-operative length of stay (days)	23 (14, 42)
Hospital length of stay (days)	31 (18, 54)

Note: RACHS-1 = Risk-Adjusted Classification for Congenital Heart Surgery; STAT = The Society of Thoracic Surgeons-European Association for Cardio-Thoracic Surgery. Continuous and categorical variables presented as median (IQR) and frequency (%), respectively

a Respiratory = Bronchopulmonary dysplasia, single lung, mechanical ventilation; Renal = Kidney dysfunction or failure requiring dialysis; Infectious = RSV, sepsis.

b Death during hospitalization or before end of 30th post-operative day if discharged from hospital.

**Table 2:** Incidence of post-operative outcomes and adverse safety events

Outcome/Event	Frequency (%)
**Post-operative outcome**	
Death^a^	12 (8)
Mechanical circulatory support^b^	11 (8)
Peri-operative cardiac arrest^c^	6 (4)
Cardiac dysfunction/failure^d^	5 (4)
Respiratory failure^e^	19 (13)
Renal failure^f^	3 (2)
Composite post-operative outcome^g^	37 (26)

**Adverse safety event**	
Insulin^h^	63 (44)
Hyperglycemia^i^	109 (77)
Poor wound healing^j^	5 (4)
Infection^k^	4 (3)
Composite safety^l^	114 (80)

**Other clinical outcome**	
Operative mortality^m^	12 (8)
Major complication^n^	23 (16)

Note:

a Defined as death by discharge, but not within 36 h post-operatively.

b Defined as intra-aortic balloon pump, ventricular assistant device, extracorporeal membrane oxygenation, or cardiopulmonary support.

c Defined as unexpected perioperative (intraoperative and postoperative) cardiac arrest.

d Cardiac dysfunction defined as low cardiac output syndrome; cardiac failure defined as severe cardiac dysfunction.

e Mechanical ventilator support > 7 days, reintubation, or need for tracheostomy.

f Dialysis or hemofiltration post-operatively or at the time of discharge.

g Any of death, mechanical circulatory support, cardiac arrest, cardiac dysfunction/failure, respiratory failure, or renal failure.

h Defined as any insulin exposure within 24-h post-operatively.

i Defined as any serum glucose ≥ 200 mg/dL within 24-h post-operatively.

j Defined as sterile wound dehiscence or sternal instability.

k Defined as pneumonia, sepsis, endocarditis, or wound infection (superficial, deep, or mediastinitis).

l Any of insulin exposure, hyperglycemia, poor wound healing, or infection.

m Defined as death during hospitalization in which operation performed, even after 30 days; or after discharge from hospital, but before the end of the 30th post-operative day.

n Defined by the Society of Thoracic Surgeons as renal failure requiring dialysis, neurologic deficit persisting at discharge, arrhythmia requiring permanent pacemaker, paralyzed diaphragm/phrenic nerve injury, mechanical circulatory support, and unplanned surgical or catheter-based cardiac and noncardiac re-interventions.

## Data Availability

The datasets generated during and/or analyzed during the current study are available from the corresponding author on reasonable request.
