# Supplementary figures and images for: Using Pharmacokinetic Modeling and Electronic Health Record Data to Predict Clinical and Safety Outcomes after Methylprednisolone Exposure during Cardiopulmonary Bypass in Neonates
Source: Congenit Heart Dis. Author manuscript; Available in PMC 2023 Jul 21. (PMC10361697; doi:10.32604/chd.2023.026262)

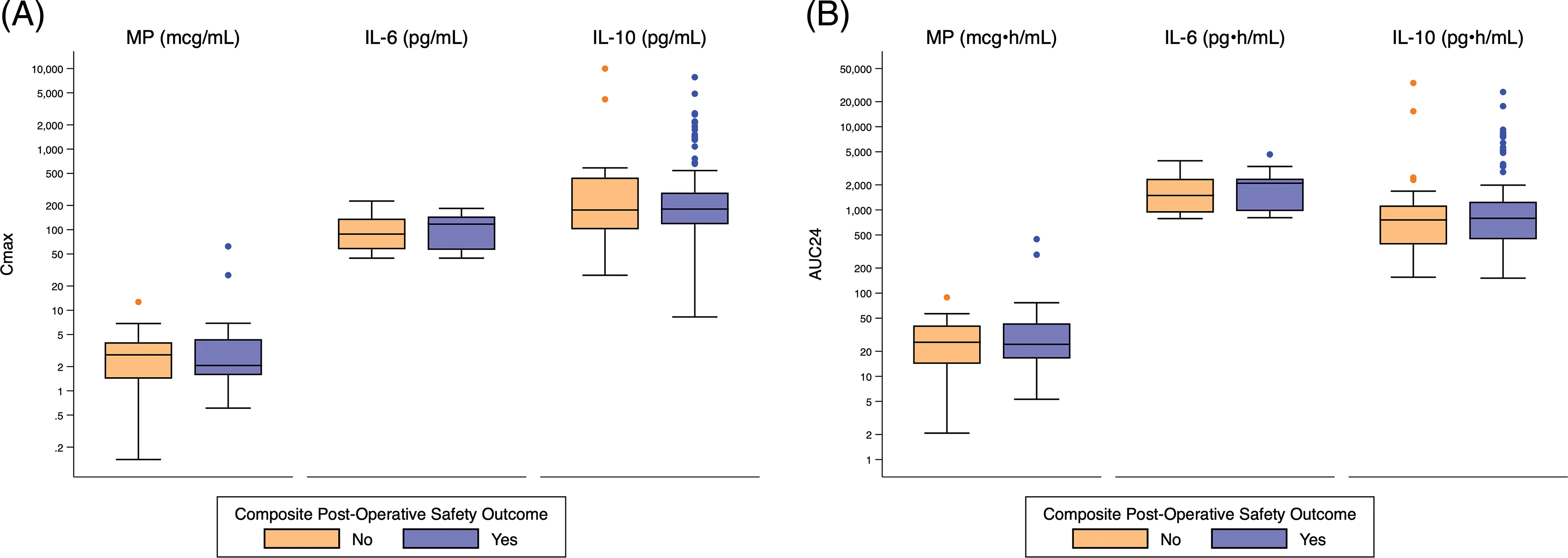

Supplement: Appendix J [file NIHMS1915879-supplement-Appendix_J.tif]

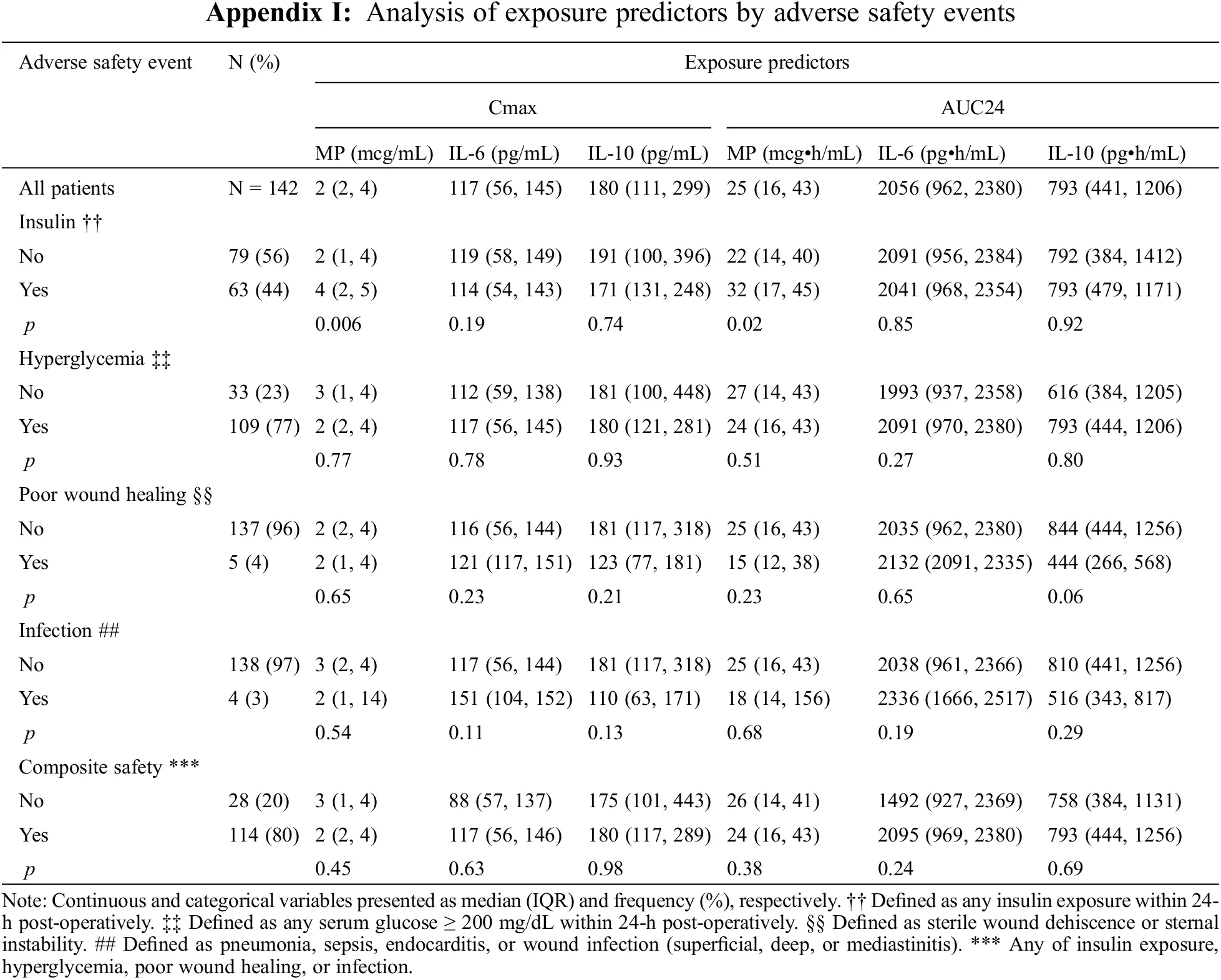

Supplement: Appendix I [file NIHMS1915879-supplement-Appendix_I.tif]

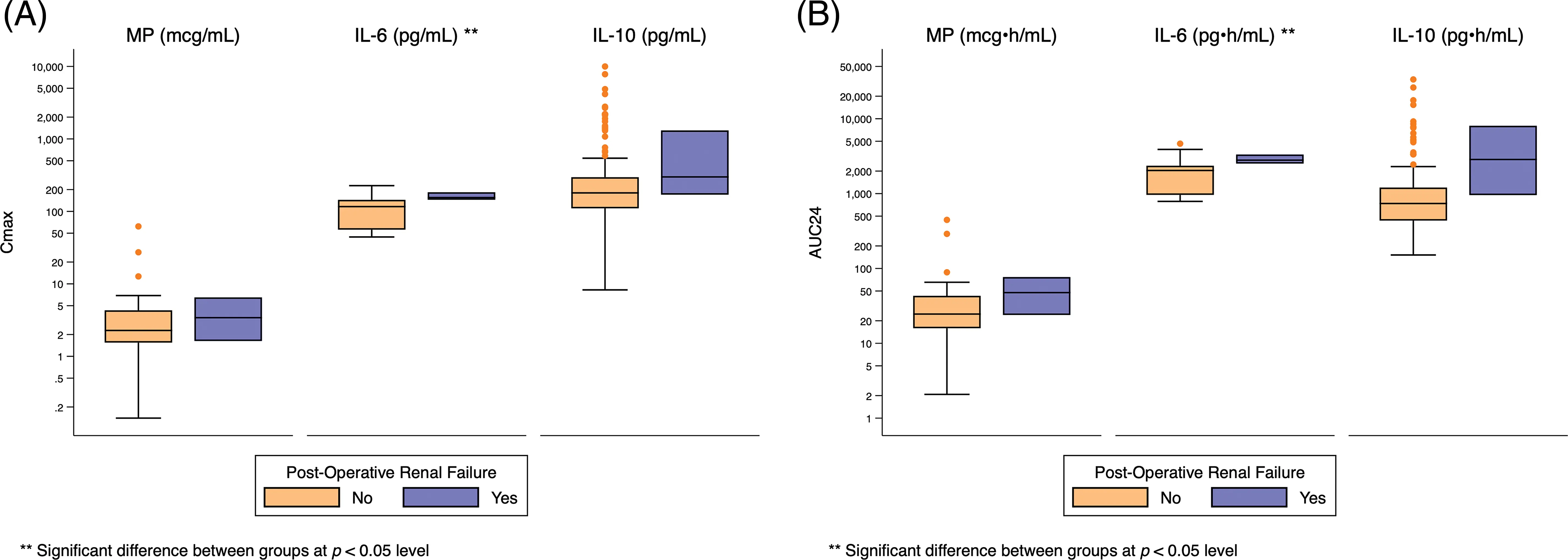

Supplement: Appendix H [file NIHMS1915879-supplement-Appendix_H.tif]

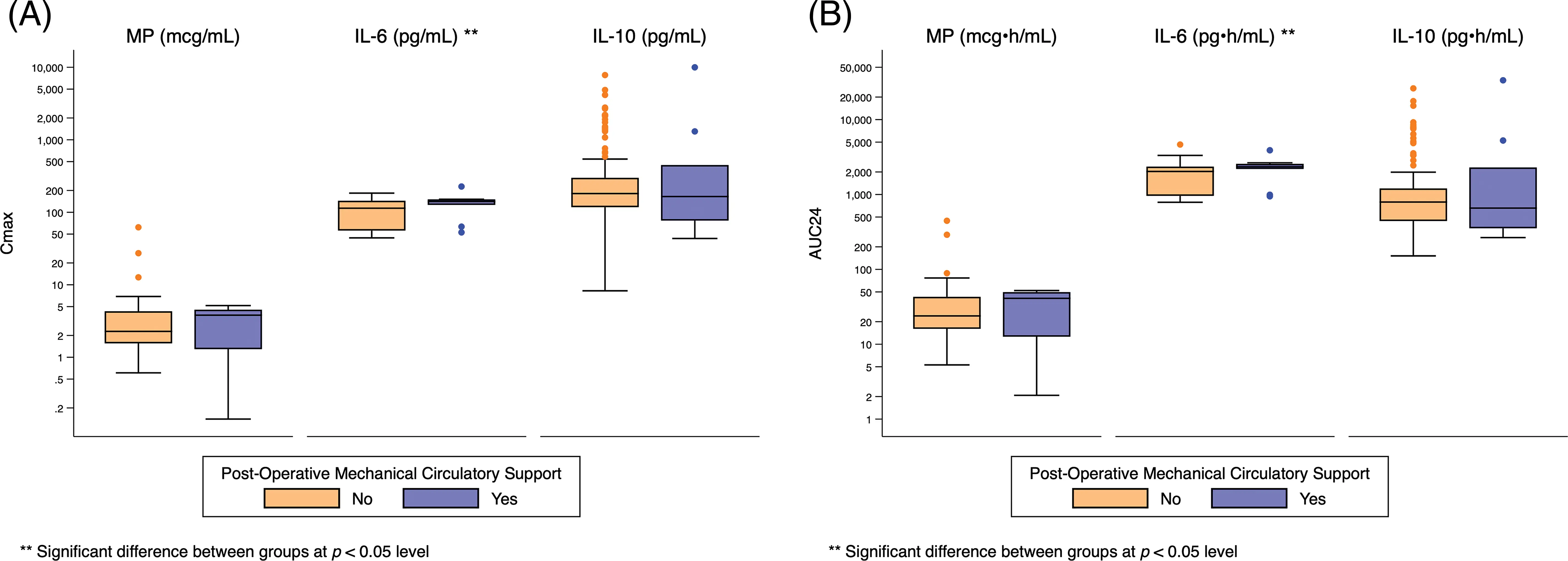

Supplement: Appendix F [file NIHMS1915879-supplement-Appendix_F.tif]

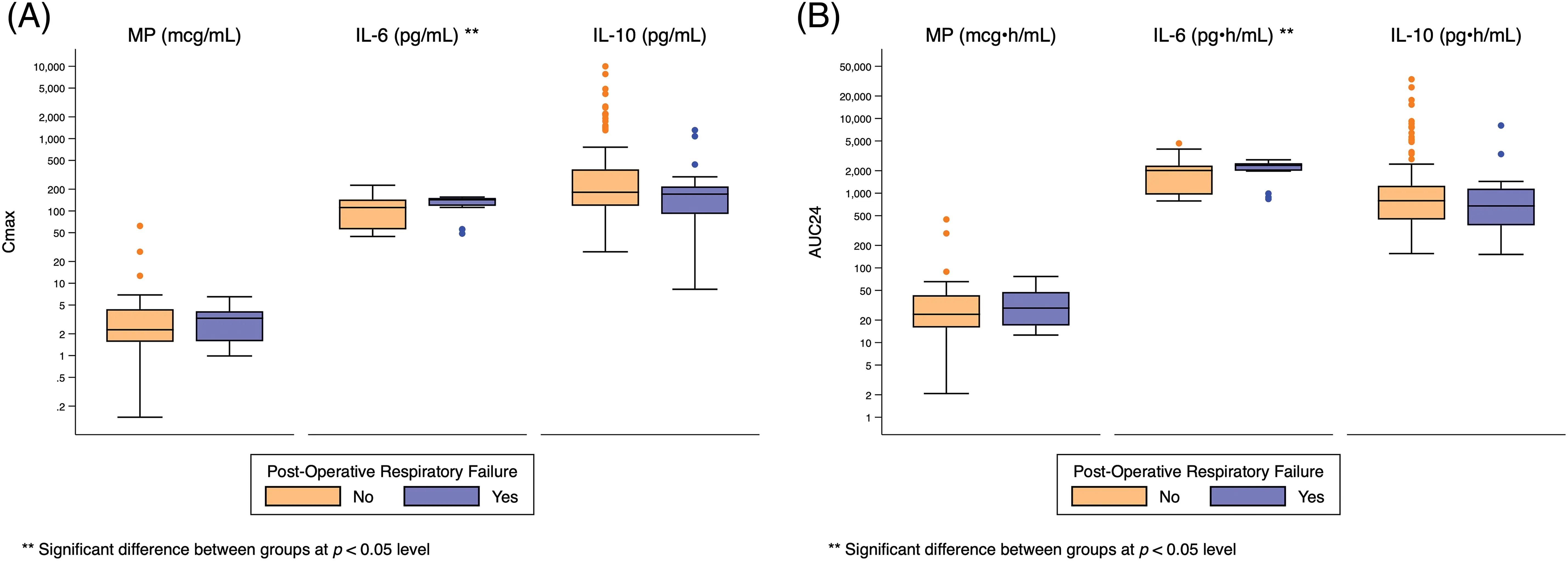

Supplement: Appendix G [file NIHMS1915879-supplement-Appendix_G.tif]

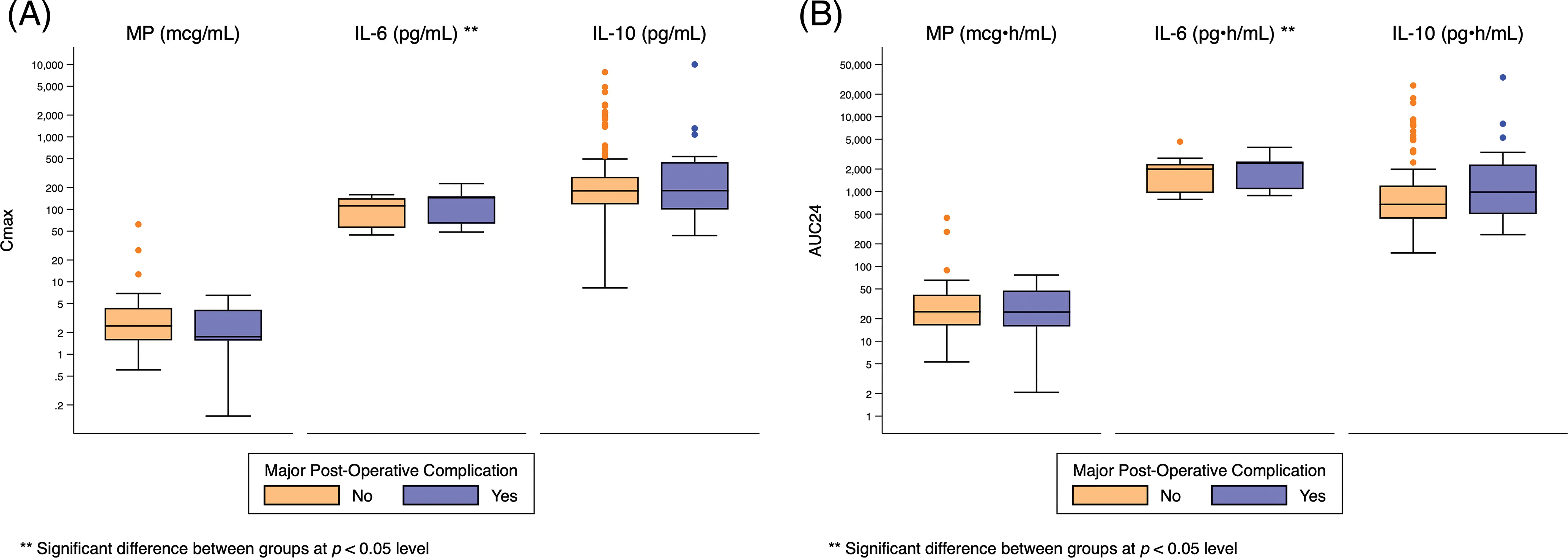

Supplement: Appendix E [file NIHMS1915879-supplement-Appendix_E.tif]

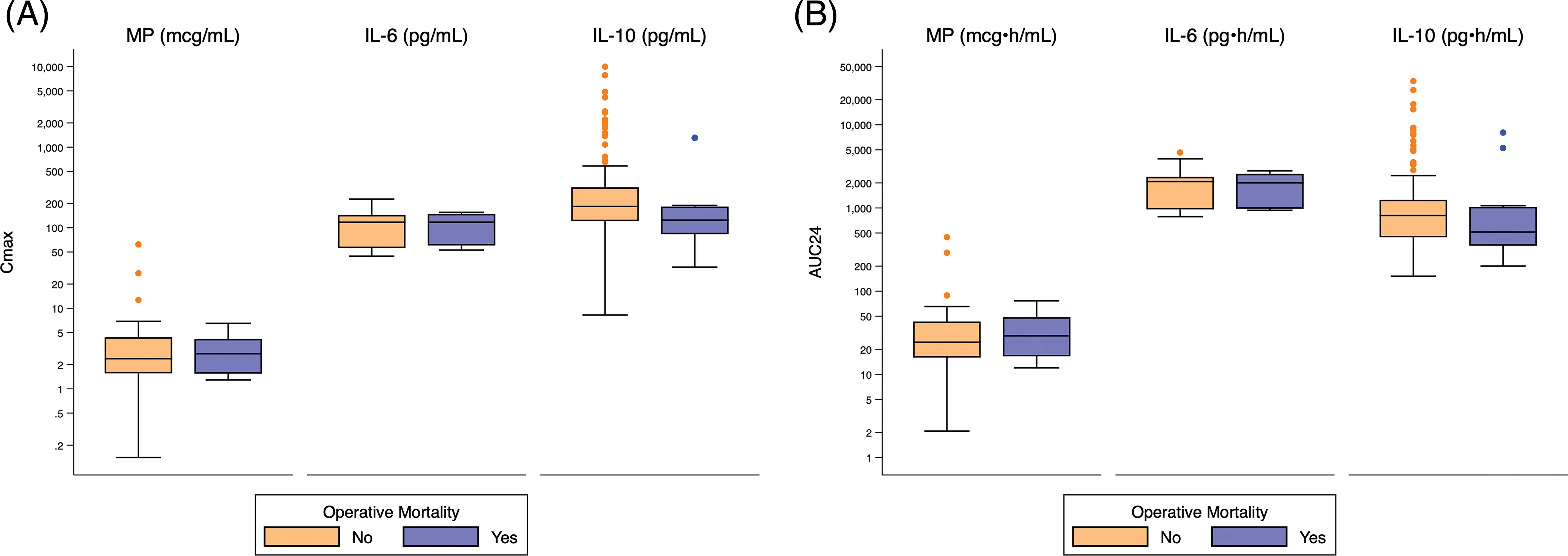

Supplement: Appendix C [file NIHMS1915879-supplement-Appendix_C.tif]

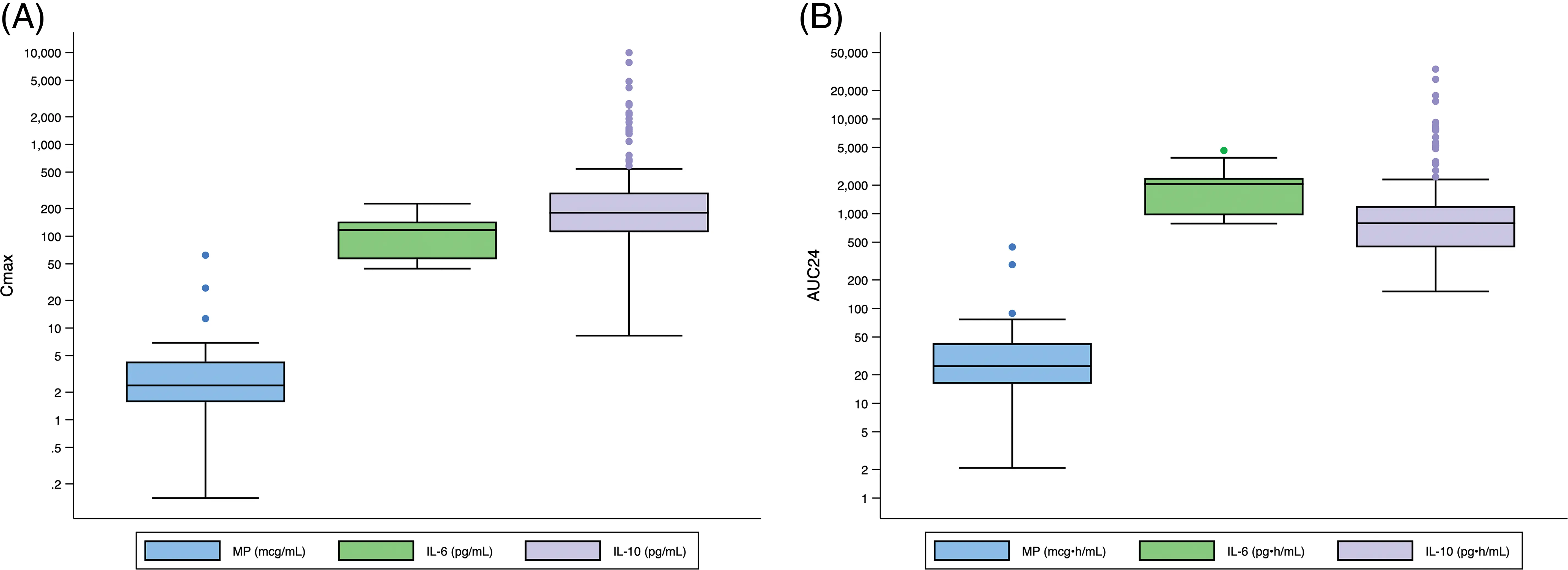

Supplement: Appendix B [file NIHMS1915879-supplement-Appendix_B.tif]

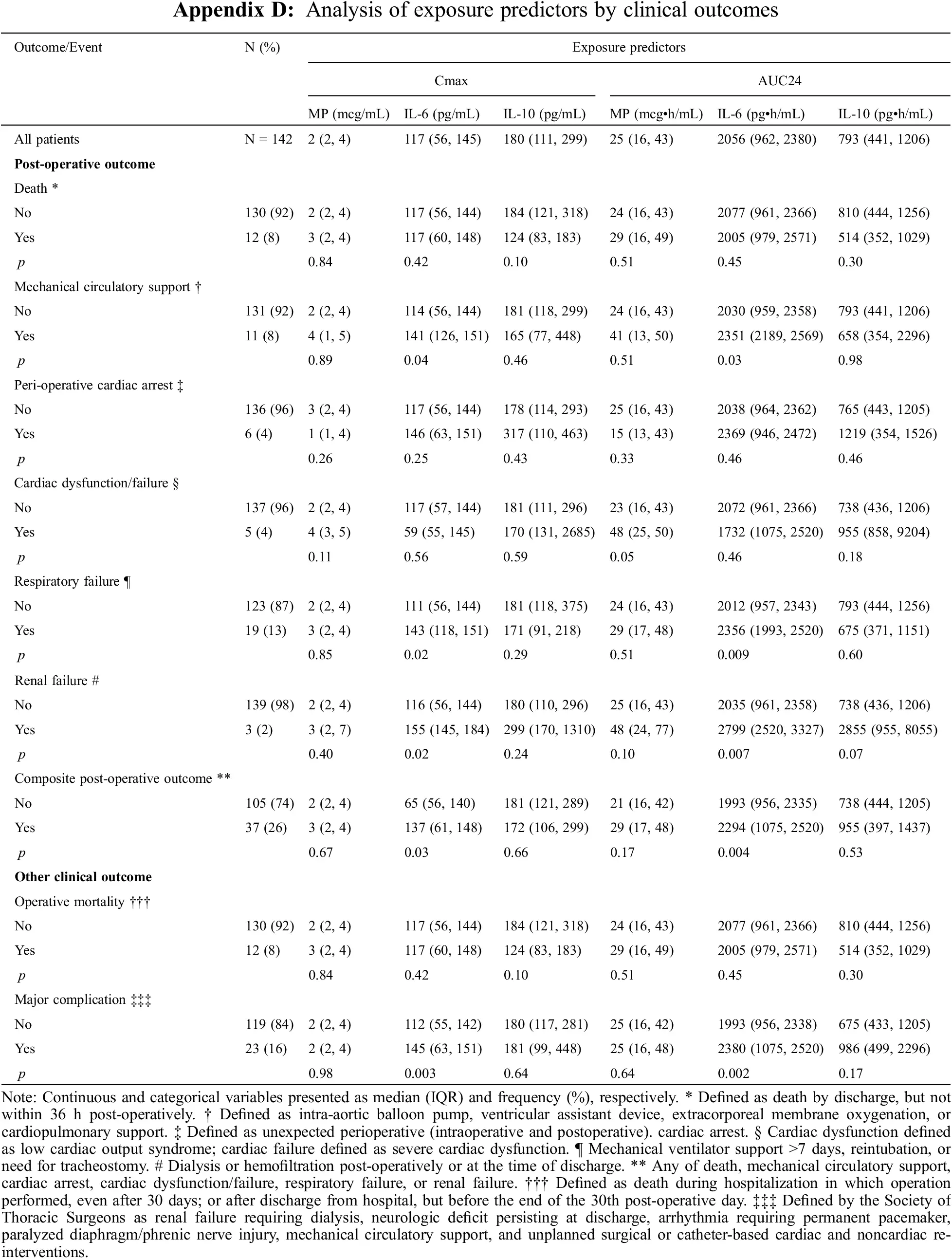

Supplement: Appendix D [file NIHMS1915879-supplement-Appendix_D.tif]
