# Supplementary material for: Using Pharmacokinetic Modeling and Electronic Health Record Data to Predict Clinical and Safety Outcomes after Methylprednisolone Exposure during Cardiopulmonary Bypass in Neonates
Source: Congenit Heart Dis. Author manuscript; Available in PMC 2023 Jul 21. (PMC10361697; doi:10.32604/chd.2023.026262)
Supplement: Appendix A [file NIHMS1915879-supplement-Appendix_A.docx]

**Appendix A. Supplementary Methods**

For change in IL-6 over time, an indirect response model with partial interaction between CPB effect and drug effect provided the best fit to the data and was selected as the base model (Eq. (S1)). Relationship between IL-6 formation and decline as well as parameterization of drug and CPB effect are shown in Eqs. (S2)–(S4), respectively. RACHS-1 score was a significant covariate for the CBP effect (CBPE) on IL-6 (Eq. (S5)):

$\frac{dIL-6}{dt}=R_{in}*\left[ \left( 1+CPBFX*\left( 1-\frac{PER}{100} \right) \right)*\left( 1-DFX \right)*STRT+CPBFX*\frac{PER}{100}+\left( 1-STRT \right)*\left( 1-DFX \right) \right]-R_{out}*IL-6$ (S1)

$R_{in}= R_{out}*IL-6_{base}$ (S2)

$DFX=\frac{\left( I_{max}*C_{P}^{HILL} \right)}{({IC}_{50}^{HILL}+C_{P}^{HILL})}$ (S3)

$CPBFX=CPBE*[1*ONCPB*CPBES+\left( 1-ENDT \right)*e^{\left( -0.693*\frac{TACPB}{CPBH} \right)}]$ (S4)

*IL-6 model CPBE = 48.6*(2.59)^RACHS-1 ≥4^*  (S5)

where R in is the zero-order rate for production of IL-6, and R out is the first-order rate for decline of IL-6. DFX and CPBFX are equations illustrating the drug and CPB effects, respectively. PER is the percent interaction between CPB and drug effects. STRT is 0 prior to start of CPB and 1 thereafter. IL-6base is the model-predicted IL-6 plasma concentration prior to the first dose of MP. Imax is the maximum fold change in the production of IL-6 as a response to drug exposure. IC50 is the methylprednisolone concentration that produces 50% of maximum attainable inhibition. HILL is the Hill coefficient. Cp is the predicted plasma concentration of methylprednisolone. CPBE is the fold change in IL-6 as a response to CPB procedure. ONCPB is a dummy indicator variable that takes the value 0 for no CPB and 1 during CPB. CPBES, used to represent the delay in CPB effect onset, is 0 for the first 30 min after start of CPB and 1 thereafter. ENDT is 0 post-CPB stop and 1 prior to CPB stop. TACPB is the time after the end of CPB. CPBH is the half-life of CPB effect. The model predicted values were 14 ng/mL for IC50, 7.9 pg/mL for IL-6base, 0.171/h for Rout, 2.53 for HILL, 48.6 for CPBE, 21.4% for percent of CPB effect not interacting with MP, 9.08 h for CPB effect half life, and 2.59 for the effect of RACHS-1 ≥ 4 on CPBE.

For change in IL-10 over time, an indirect response model with complete interaction between CPB effect and drug effect provided the best fit to the data and was selected as the base model (Eq. (S6)). Post-menstrual age (PMA) was a significant covariate for the CBPE for IL-10 (Eq. (S7)). Changes in IL-10 plasma concentrations over time were otherwise characterized using equations similar to those for IL-6 except a stimulatory Emax model (Smax) was used to account for the stimulation effect of methylprednisolone on R in for IL-10 (Eq. (S8)):

$\frac{dIL-10}{dt}=R_{in}*\left( 1+CPBFX \right)*\left( 1+DFX \right)-R_{out}*IL-10$ (S6)

*IL – 10 model CPBE = 45.7*(PMA/40)^14.8^*  (S7)

$DFX=\frac{\left( S_{max}*C_{P}^{HILL} \right)}{({IC}_{50}^{HILL}+C_{P}^{HILL})}$ (S8)

Model predicted values were 2.28 for Smax, 58.2 ng/mL for SC50, 1.52 pg/mL for IL-10base, 0.542/h for Rout, 3.58 for HILL, 45.7 for CPBE, and 14.8 for effect of PMA on CPBE.

We used the nonlinear mixed-effect modeling software NONMEM and simulated concentrations of MP, IL-6, and IL-10 at both maximum (Cmax) and integrated concentration over 24 h post-operatively (AUC24). AUC24 was calculated by Eq. (S9):

${AUC}_{24}=\int_{t1}^{t1+24} Cdt$ (S9)

where C is concentration, t1 is time of CBP start relative to first dose of MP, and t is time after the first dose.
